# Simulation Training in Neuroangiography—Validation and Effectiveness

**DOI:** 10.1007/s00062-020-00902-5

**Published:** 2020-04-17

**Authors:** Kornelia Kreiser, Lea Ströber, Kim G. Gehling, Frederick Schneider, Stefan Kohlbecher, Christian M. Schulz, Claus Zimmer, Jan S. Kirschke

**Affiliations:** 1 Department of Diagnostic and Interventional Neuroradiology, Klinikum rechts der Isar, Technische Universität München, Ismaninger Str. 22, 81675 Munich, Germany; 2 Department of Anesthesiology, Klinikum rechts der Isar, Technische Universität München, Munich, Germany; 3 EyeSeeTec GmbH, Munich, Germany

**Keywords:** Neuroradiology, Training effect, Validity, Eye tracking, Work load

## Abstract

**Purpose:**

Simulators are increasingly used in the training of endovascular procedures; however, for the use of the Mentice vascular interventional system trainer (VIST) simulator in neuroradiology, the validity of the method has not yet been proven. The study was carried out to test the construct validity of such a simulator by demonstrating differences between beginner and expert neurointerventionalists and to evaluate whether a training effect can be demonstrated in repeated cases for different levels of experience.

**Methods:**

In this study 4 experts and 6 beginners performed 10 diagnostic angiographies on the VIST simulator (Mentice AB, Gothenburg, Sweden). Of the cases four were non-recurring, whereas three were repeated once and ten subjects performed all tasks. Additionally, another expert performed only five non-recurring cases. The simulator recorded total time, fluoroscopy time, amount of contrast medium and number of material changes. Furthermore, gaze direction and heart rate were recorded, and subjects completed a questionnaire on workload.

**Results:**

Beginners and experts showed significant differences in total duration time, fluoroscopy time and amount of contrast agent (all *p* < 0.05). Gaze direction, dwell time and heart rate were similar between both groups. Only beginners improved during training with respect to total duration time, fluoroscopy time and amount of contrast agent. If a case was previously known to them, the total duration and fluoroscopy time were significantly shortened (*p* < 0.001).

**Conclusion:**

This study demonstrated both the construct validity of a diagnostic neuroangiography simulator as well as a significant training effect for beginners. Therefore, in particular beginner neurointerventionalists should use such simulation tools more extensively in their initial training.

## Background/Introduction

The number of endovascularly treated patients suffering from stroke or aneurysms has steadily increased over the last few years [1]. In 2015, five large randomized stroke trials established the superiority of mechanical thrombectomy in combination with intravenous recombinant tissue plasminogen activator (i.v. rt-PA) over i.v. rt-PA alone [2–6]. Since then, the number of treated patients has risen continuously. Moreover, the time window has been recently broadened [7, 8], which will further increase the number of patients eligible for interventional treatment. Although mechanical thrombectomy is performed in more and more patients at centers with great expertise, demand is increasing for an on-call service around the clock at multiple smaller centers. Hence, a sufficient number of interventional neuroradiologists must be trained at a high level. Using high-fidelity simulators offers the possibility of structured training without endangering patients [9, 10]. Although the first prototypes of simulators specifically for neuroradiological applications were developed many years ago [11, 12], they are not yet widely used in neuroradiology. After proof of face and content validity, based on expert opinion [13] the next crucial step to establish such a new approach includes the demonstration of its construct validity, i.e. beginners and advanced users can be distinguished via the parameters measured by the simulator. Only when this prerequisite is met should such a simulator be used for training interventionalists. Concerning interventional procedures, construct validity was previously shown for renal [14, 15] and cardiac procedures [16, 17] as well as for carotid stenting [18, 19]. After the validity or neuroangiographies of another make, the ANGIO Mentor Express (Simbionix, Cleveland, OH, USA) has already been proven [20], the present study aimed at establishing the construct validity of the Mentice VIST simulator; however, construct validity is not directly related to the effectiveness of a simulator. To be used as an effective training tool in practice, improvements in psychomotor skills should be measurable [21, 22]. Therefore, the second aim of the study was to quantify improvements of different quality measures of a diagnostic neuroangiography, resulting from repeated simulator training. Of interest was, in addition to an already confirmed training effect for beginners and intermediates [10, 20, 23, 24], whether this impact could also be demonstrated for experts.

## Material and Methods

### Hardware

**Fig. 1:**
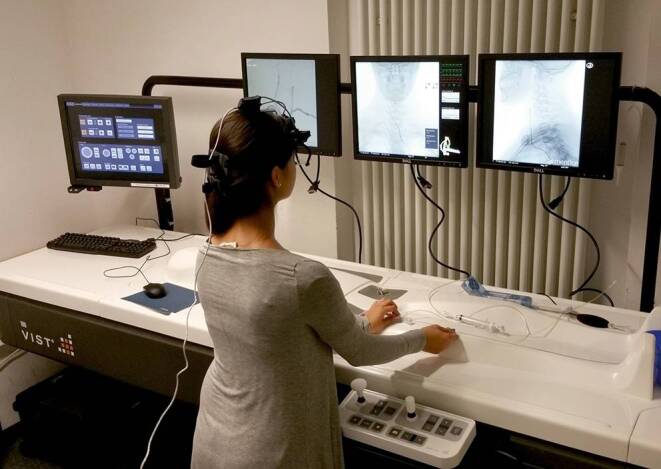
Simulation set-up with VIST LAB and eye tracking camera (heart rate belt covered by shirt)

All neuroangiographic simulations were performed with the vascular intervention system trainer VIST C from Mentice (Mentice AB, Gothenburg, Sweden), integrated in the VIST LAB. This is a stationary unit whose surface is designed in the form of a patient silhouette (Fig. 1). The VIST LAB consists of a control screen with a touch screen function for selecting scenarios and materials, plus three additional monitors. On two of them, the examiner can follow the live image on two planes and on the third monitor can scroll through the recorded series. A laptop and the simulator itself are positioned under the surface. The only visible detail of the simulator is an insertion sheath at the height of the manikin’s groin, which serves as the access point to the simulator for all materials. Pushing, pulling, and rotating movements of the introduced wires and catheters are detected by three sensors, which can also generate resistance in the sense of force feedback. These movements are transferred into a virtual patient anatomy, consisting of arteries and bones, and displayed on the screens in real time. A foot switch with two pedals, a panel with several options for controlling the virtual C-arms and other settings (shutter, zoom, etc.), and a tube with a syringe for the injection of air, simulating the injection of contrast medium, are also connected to the simulator.

Parameters stored by the device are total duration of the procedure, total fluoroscopy time, number of series, total series time, amount of contrast agent, and number of material changes.

### Endovascular Devices

Real angiographic materials, such as a hydrophilic wire (0.035″ Glidewire, Terumo, Somerset, NJ, USA) and a 5F vertebralis diagnostic catheter (VER, Cordis, Santa Clara, CA, USA) were used. All terminal curvatures had been cut off as required by the manufacturer of the simulator.

### Software

The source data of magnetic resonance (MR) angiographies of real patients with different anatomical types of the aortic arch were segmented semi-automatically by using IntelliSpacePortal (Philips, Best, The Netherlands). The resulting 3D model was imported to the simulator as a Stereolithography (STL) file. The integrated Case-it software module (Mentice AB, Gothenburg, Sweden) connected the model of the aortic arch and the supra-aortic branches to a template of the descending aorta and the iliac arteries down to the superficial femoral artery.

### Accessories

An eye-tracking camera (EyeSeeCam Sci, EyeSeeTec GmbH, München, Germany) was used to record the viewing direction of the test persons. The pulse rate was recorded with a heart rate belt (Zephyr™ BioModule™ Sensor, BioHarnessTM 3, Medtronic, Dublin, Ireland). Free MATLAB®-based (MathWorks®, Natick, MA, USA) ARTiiFACT software [25] was used to evaluate the mean heart rate. The whole set-up is presented in Fig. 1.

### Test Persons

Participants were recruited from the radiology and the neuroradiology departments and divided into two groups. The expert group consisted of five physicians with advanced angiography experience from more than 100 cerebral angiographies each. One of them only completed the first five cases and then left the study. These data could only be included in the analysis of validity, but not in the evaluations of the training effect. The beginner group consisted of one medical student without any experience in angiography, three neuroradiological residents with little neuroangiographic experience (<30) and two radiological residents with exclusively peripheral angiographic experience.

The local ethics committee approved the study (172/14), and each participant provided informed consent before participating.

### Study Design

Each participant had to complete ten cases. Every case consisted of a complete cerebral angiography in one patient, i.e., the selective probing and visualization of the internal carotid artery, external carotid artery, and vertebral artery with their respective dependent branches on both sides. The ten cases were split in two study parts, where the first part included five unknown cases and the second part included two new cases and three repeated cases from the first part. In detail, the first case was repeated in case six, the fourth case in case nine and the fifth case in case ten. All cases were classified into five levels of difficulty by a neuroradiologist not participating in the study. The classification was based on the following criteria: level 1 was easy to perform with a VER catheter without using a roadmap due to standard anatomy; level 2 was also feasible with a VER catheter, but a roadmap was required; level 3 included an arterial norm variant, i.e., the left vertebral artery originated directly from the aortic arch. In level 4, a sidewinder catheter was required to solve the task. The highest level 5 required advanced catheter maneuvers and changes, plus precise knowledge of the vessel anatomy, variants, and possible pathologies. In each part of the study there was one case of each difficulty level. The participants were informed neither about the level nor about whether the presented case was a known or unknown one.

Before the first session, a technical introduction to the simulator was given, together with a shortened exercise session on a case that was not part of the study. The participant was equipped with a heart rate belt and a head-mounted camera. The individual simulation sessions were limited to a maximum of 2h to avoid any possible fatigue effects. The operation of the simulator beyond the catheter guidance was the responsibility of a student. Each angiography was started with a hydrophilic guidewire (35° curvature) and VER as standard, but the material could be changed at any time on request. At the end of each case, the investigators filled out a questionnaire to record the subjectively perceived workload (NASA-TLX, National Aeronautics and Space Administration Task Load Index, German translation, [26]) as rating with respect to mental demands, physical demands, temporal demands, satisfaction with performance, effort, and frustration. Each rating scale ranged from 0 to 20.

### Statistics

Statistical analysis was performed by descriptive and exploratory statistics. Ordinal data (number of series, number of material changes, results of NASA-TLX) and metric data (total duration, total fluoroscopy time, total series time, amount of contrast agent, mean heart rate and percentage of gaze direction lower than 30°) were compared by using a median test or a Mann-Whitney U‑test, respectively. Ordinal data are presented in median (interquartile range, IQR), metric data are presented as mean (± standard deviation, SD) if not stated differently. A *p*-value < 0.05 was considered as statistically significant. All analyses were performed using IBM SPSS Statistics, Version 23.0 (IBM, Armonk, NY, USA).

## Results

### Proof of Validation

Comparative results of the experts and beginners of all 10 cases are listed in Table 1. Total duration time, total fluoroscopy time and amount of contrast agent differed significantly. With respect to the subjective task requirements, significant differences were observed in the assessment of satisfaction and effort. Beginners were more dissatisfied and had to make greater efforts than the experts. Nevertheless, heart rate and viewing direction did not differ between experts and beginners in the cases.

**Table 1:** Comparison of experts and beginners in a summary of all 10 simulations (one expert only performed simulations 1–5)

	Simulations 1–10				
	Experts (*n* = 5)		Beginners (*n* = 6)		*p*-value
*Simulation data:*	*Mean*	*±SD*	*Mean*	*±SD*	*–*
Total duration (min)	19.65	7.73	31.96	15.07	***p*** **≤** **0.001**
Fluoroscopy time (min)	11.91	4.59	18.78	7.72	***p*** **≤** **0.001**
Total time of series (min)	1.90	0.80	1.75	0.79	*p* = 0.269
Contrast agent (ml)	52.04	21.31	67.20	30.82	***p*** **=** **0.013**
	*Median*	*IQR*	*Median*	*IQR*	–
Number of series	16	10	16.5	9	*p* = 0.976
Number of material changes	1	1	2	2	*p* = 0.056
*NASA-TLX (range 1–20):*	*Median*	*IQR*	*Median*	*IQR*	*–*
Mental demand	9.5	8	10.5	7	*p* = 0.795
Physical demand	4.5	4	5	4	*p* = 0.780
Temporal demand	7.5	7	6	6	*p* = 0.324
Satisfaction with performance	17	4	15	6	***p*** **=** **0.008**
Effort	11	7	15	5	***p*** **≤** **0.001**
Frustration	6	4	5	5	*p* = 0.095
*Physiological data:*	*Mean*	*±SD*	*Mean*	*±SD*	*–*
Heart rate (beats per minute/bpm)	79.00	8.92	81.47	13.86	*p* = 0.669
Viewing direction (%)^a^	29.38	14.40	30.08	14.60	*p* = 0.502

Median and IQR for number of series, number of material changes and results of NASA-TLX, *p*-values of mediantest <0.05 are in bold type. Mean for total duration, fluoroscopy time, total time of series and amount of contrast agent, heart rate and gaze direction, *p*-values of Mann Whitney U‑test <0.05 are in bold type

^a^Percentage of deviation of more than 30° from the horizontal view downwards.

### Proof of Training Effectiveness

**Fig. 2:**
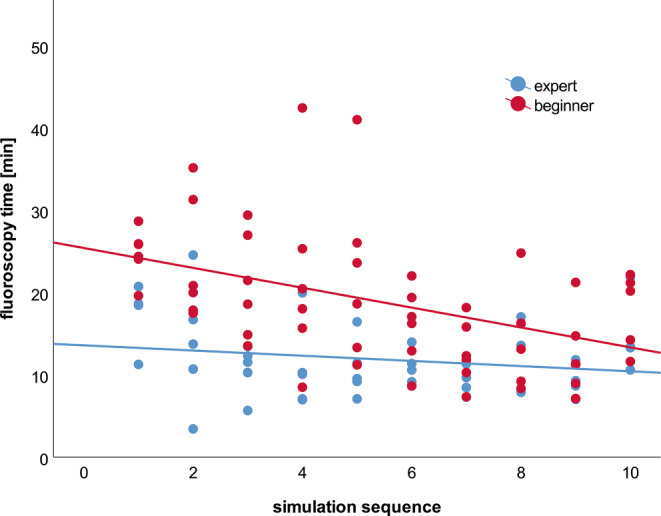
Fluoroscopy time of beginners (*red*) and experts (*blue*) with corresponding regression line during all 10 simulations. Cases 1, 4 and 5 are repeated (in same order) in cases 6, 9 and 10 respectively

In a comparison of the first five cases with the last five cases participants improved significantly in terms of total duration, fluoroscopy time and perceived effort (total duration 31.94 min (SD 16.41 min) to 21.15 (SD 7.18 min), *p* < 0.001; fluoroscopy time 18.15 min (SD 8.55 min) to 13.42 min (SD 4.87 min), *p* = 0.003; effort [median] 15 (IQR 7) to 13 (IQR 7), *p* = 0.031); however, when results are separated for experts and beginners (Table 2), then this effect remained significant only in beginners. Fig. 2 visualizes the decrease of fluoroscopy time during the sequence of the training, although the degree of difficulty increased from 1 to 5 and 6 to 10 respectively. In addition, the beginners became more satisfied with their performance during the course of training. Similar differences were found when comparing unknown cases with known cases, where total duration time and fluoroscopy time differed significantly and participants were more satisfied with their performance (total duration 35.91 min (SD 16.03 min) to 22.73 min (SD 6.37 min), *p* < 0.001; fluoroscopy time 20.66 min (SD 7.90 min) to 14.39 min (SD 5.19 min), *p* = 0.003; satisfaction with performance [median] 15.5 (IQR 5) to 16 (IQR 3), *p* = 0.022).

**Table 2:** Comparison of experts and beginners of simulations 1–5 and 6–10 separately

	Experts					Beginners				
	Simulation (*n* = 5) 1–5		Simulation 6–10 (*n* = 4)		*p*-value	Simulation 1–5 (*n* = 6)		Simulation 6–10 (*n* = 6)		*p*-value
*Simulation data:*	*Mean*	*±SD*	*Mean*	*±SD*	*–*	*Mean*	*±SD*	*Mean*	*±SD*	*–*
Total duration (min)	21.88	8.79	16.99	5.29	***p*** **=** **0.045**	40.00	16.73	23.92	6.99	***p*** **≤** **0.001**
Fluoroscopy time (min	12.49	5.27	11.23	3.61	*p* = 0.465	22.68	7.98	14.87	5.10	***p*** **≤** **0.001**
Total time of series (min)	1.99	0.84	1.79	0.76	*p* = 0.588	1.84	0.79	1.67	0.80	*p* = 0.492
Contrast agent (ml)	54.05	22.41	49.62	20.20	*p* = 0.423	75.82	34.14	58.58	24.75	***p*** **=** **0.045**
	*Median*	*IQR*	*Median*	*IQR*	*–*	*Median*	*IQR*	*Median*	*IQR*	***–***
Number of series	17	9	16	12	*p* = 0.978	17	6	16	11	*p* = 0.439
Number of material changes	1	1	1	2	*p* = 0.839	2	2	1.5	1	*p* = 0.267
*NASA-TLX* *(range 1–20):*	*Median*	*IQR*	*Median*	*IQR*	*–*	*Median*	*IQR*	*Median*	*IQR*	–
Mental demand	10	9	10	8	*p* = 0.762	11.5	9	10	6	*p* = 0.439
Physical demand	4	4	5	6	*p* = 0.762	4	2	5.5	5	*p* = 0.434
Temporal demand	8	8	7	7	*p* = 0.762	6.5	7	6	5	*p* = 1.000
Satisfaction with performance	17	2	16	4	*p* = 0.226	13.5	6	16	2	***p*** **=** **0.009**
Effort	11.5	7	11	6	*p* = 0.978	15.5	3	13.5	5	*p* = 0.796
Frustration	5.5	5	7	3	*p* = 0.392	5	7	4	5	*p* = 0.595
*Physiological data:*	*Mean*	*±SD*	*Mean*	*±SD*	*–*	*Mean*	*±SD*	*Mean*	*±SD*	*–*
Heart rate (bpm)	78.06	7.74	80.14	10.25	*p* = 0.220	84.07	14.88	78.86	12.47	*p* = 0.322
Viewing direction (%)^a^	27.47	12.00	31.68	16.69	*p* = 0.377	31.55	18.31	28.60	9.70	*p* = 0.982

Median and IQR for number of series, number of material changes and results of NASA-TLX, *p*-values of mediantest <0.05 are in bold type. Mean for total duration, fluoroscopy time, total time of series and amount of contrast agent, heart rate and gaze direction, *p*-values of Mann Whitney-U-Test <0.05 are in bold type

^a^Percentage of deviation of more than 30° from the horizontal view downwards.

Again, these differences remained significant only for beginners (Table 3). Neither heart rate, nor viewing direction differed in experts or beginners between known and unknown cases.

**Table 3:** Comparison of experts and beginners regarding unknown and known cases

	Experts (*n* = 4)					Beginners (*n* = 6)				
	Unknown cases		Known cases		*p*-value	Unknown cases		Known cases		*p*-value
*Simulation data:*	*Mean*	*±SD*	*Mean*	*±SD*	*–*	*Mean*	*±SD*	*Mean*	*±SD*	–
Total duration (min	20.90	8.42	16.34	4.19	*p* = 0.131	35.91	16.03	22.73	6.37	***p*** **≤** **0.001**
Fluoroscopy time (min)	12.36	5.03	10.71	2.98	*p* = 0.397	20.66	7.90	14.39	5.19	***p*** **=** **0.003**
Total time of series (min)	1.93	0.83	1.83	074	*p* = 0.765	1.81	0.78	1.64	0.82	*p* = 0.529
Contrast agent (ml)	52.84	22.04	49.89	19.98	*p* = 0.745	71.95	30.90	56.12	28.43	*p* = 0.058
	*Median*	*IQR*	*Median*	*IQR*	*–*	*Median*	*IQR*	*Median*	*IQR*	*–*
Number of series	17	12	16	7	*p* = 0.878	17	7	16	11	*p* = 0.398
Number of material changes	1	1	1	2	*p* = 0.673	2	2	1	1	*p* = 0.183
*NASA-TLX* *(range 1–20):*	*Median*	*IQR*	*Median*	*IQR*	*–*	*Median*	*IQR*	*Median*	*IQR*	*–*
Mental demand	10	9	8	8	*p* = 0.735	11.5	9	8	6	*p* = 0.398
Physical demand	4	4	5	6	*p* = 0.735	5	4	5	4	*p* = 0.865
Temporal demand	8	7	7	7	*p* = 0.735	6.5	7	6	5	*p* = 0.910
Satisfaction with performance	17	3	15.5	4	*p* = 0.338	14	6	16	2	***p*** **=** **0.022**
Effort	12.5	5	9.5	6	*p* = 0.406	15.5	3	13	7	*p* = 0.611
Frustration	6	5	6.5	2	*p* = 0.975	5	8	4	3	*p* = 0.164
*Physiological data:*	*Mean*	*±SD*	*Mean*	*±SD*	*–*	*Mean*	*±SD*	*Mean*	*±SD*	*–*
Heart rate (bpm)	78.77	8.41	79.61	10.54	*p* = 0.524	82.4	14.30	79.09	12.85	*p* = 0.594
Viewing direction (%)^a^	27.75	13.36	33.73	16.38	*p* = 0.234	30.79	16.43	28.41	9.23	*p* = 0.949

Median and IQR for number of series, number of material changes and results of NASA-TLX, *p*-values of median test <0.05 are in bold type. Mean for total duration, fluoroscopy time, total time of series and amount of contrast agent, heart rate and gaze direction, *p*-values of Mann Whitney U‑test <0.05 are in bold type

^a^Percentage of deviation of more than 30° from the horizontal view downwards

## Discussion

In this study it was possible to prove the construct validity of a neuroangiographic simulator by demonstrating significant performance differences between beginners and experts. We were furthermore able to show the effectiveness of such a simulator by directly measuring significant improvements in psychomotor skills of beginners in cases derived from real patient anatomy.

This is in line with previous studies on different scenarios, such as carotid artery stenting [13, 18] and several infra-aortic applications [9, 17] and addresses one major concern regarding neuroangiographic simulations: In 2008, Carroll and Messenger stated that “medical simulation has made the transition from an experimental technology to the clinical world”, and that “perhaps the most pressing issue […] regarding medical simulation is validation” [27].

To prove the construct validity, we had clearly separated groups of beginners with a maximum of 30 cerebral angiographies performed and of experts with at least 100 procedures. The simulator data for all 10 procedures evidently demonstrated this subdivision. As previously confirmed for cardiac angiography [28], beginners need more time to find and examine the target vessels. Thus, total duration and fluoroscopy time differed significantly. Beginners more often produced roadmaps; accordingly, the total amount of contrast agent was significantly higher for beginners. The fact that the simulator only shows the contrast of the arteries and contains no parenchyma or the venous phase may serve as a reason for the missing difference in the duration of the series. Thus, in this parameter no difference was present between experts and beginners. Overall, these results contradict the findings of Nguyen et al. who were only able to identify the amount of contrast agent as a distinguishing feature for the experience level [20]. A possible reason could be their small number of 2 compared tasks, while our study design consisted of 10 procedures.

Observations of gaze direction, blink frequency, pupil size and dwell time are recognized means of examining attention and cognitive stress [29]. Based on such data Richstone et al. were able to distinguish unequivocally between beginners and experts in surgical laparoscopy [30], but for endovascular cardiac interventions, Currie et al. found hardly any differences [31]. The assumption that experts would turn their gaze less often away from the X‑ray screen could therefore not be confirmed by them. The present study focused on the direction of each subject’s gaze; no differences between the two groups could be observed.

Heart rate was recorded, as it was assumed that it would differ between various levels of workload and thus between beginners and experts according to the effort made. In previous work on anesthesiologists, Martin et al. as well as Weinger et al. found significant differences in several heart rate parameters, including mean heart rate, during different phases of an anesthetic procedure [32, 42]. In a later work of the first group on pre-hospital emergency medicine, heart rate variability discriminated better between different levels of workload compared to the mean heart rate [33]. Currie et al. also measured numerous parameters such as heart rate variability, electrodermal activity and skin temperature in physicians undertaking cardiac endovascular procedures but could not find differences between experience levels [31]. Hence, mean heart rate seems to be an unreliable predictor, matching the results of our study, where it was not suitable to distinguish between beginners and experts.

The NASA-TLX is a frequently used tool to assess subjective workload, especially in anesthesia and the field of emergency care [34], but also in other areas, e.g. radiotherapy [35] or flight simulation [36]. In comparison with the previously mentioned complex technical tools, the NASA-TLX offers an easy and fast method for recording the workload. The modified version Raw-TLX (RTLX), without the weighting process of the subscales, is even easier to use [37]. In a study on surgical robotics, differences were found in NASA RTLX by examiners with different experience levels [38], just as in our participants. Apparently, there are no data on whether NASA RTLX changes differently through training of beginners compared to experts. The satisfaction of our participants only increased when the other measurable parameters also improved, which was only true for beginners. We therefore assume that the simulator is well suitable to give adequate feedback on one’s own performance.

An increase in performance with respect to the two essential parameters of total duration and fluoroscopy time has been demonstrated in our study for beginners and confirmed the results of Spiotta et al. and Zaika et al. [10, 24]. Among our participants, however, this effect could not be observed among experts. Thus, not every experience level benefits from simulator training that focuses exclusively on diagnostic angiography. This supports the assumption that in the best case skills are learned that an expert already possesses. For beginners, the training effect can be objectively read from the metrics of the simulator, as known for peripheral endovascular procedures [14, 39]. In addition, we have shown that repeated exercises of the same cases are helpful for beginners not only to increase their metric values, but also to increase their own satisfaction. Spiotta et al. also noted an increase in confidence in the acquisition of skills, both in terms of knowledge of the anatomy and the technique of vessel selection [23]. Not least for this reason, several centers have now begun to implement a structured milestone-based curriculum and propose to integrate simulation training into formal neuroendovascular training [23, 40]

When demonstrating a training effect, it is always difficult to distinguish the extent to which a real gain in specific psychomotor skills overlaps with increasing familiarity with the operation of the simulator [39]. An improvement merely by habituation would be recognizable in all subjects; however, since only the group of beginners improved their performance relevantly, this effect seems to be negligible here.

Notably, in the RTLX evaluation, the degree of frustration of the experts slightly increased during training, whereas it decreased among the beginners (both not significant). Comments from the participants showed that individual experts were disturbed by the differences between simulation and reality, whereas beginners were not. The participants of a simulator training should therefore be informed in advance with respect to the differences that can be expected in the behavior of the simulator in relation to reality. Otherwise, they approach the training with a variety of ideas and demands, and frustration and anger can easily arise [1].

## Limitations

Construct validity should distinguish not only between beginners and experts, but ideally also between various levels of experience [16]. In our study, the small number of test persons prevented further division of study participants. Also, the number of cases, in particular of repeated cases was limited in this study. A higher number of cases might also show differences in expert neuroradiologists.

## Conclusion

Construct validity of a high-tech simulator could be demonstrated for diagnostic neuroangiography and especially beginners showed a measurable training effect through repeated practice. Further studies should demonstrate the benefit of such simulation training for the patient.

## Abbreviations

bpm: Beats per minute
F: French
IQR: Interquartile range
NASA TLX: National Aeronautics and Space Administration Task Load Index
RTLX: RAW TLX (unweighted task load index)
rtPA: Recombinant tissue plasminogen activator
SD: Standard deviation
STL: Stereolithography (file format)
VER: Vertebralis catheter
